# Trajectory of *Plasmodium falciparum* Molecular Markers of Amodiaquine Resistance in São Tomé and Príncipe

**DOI:** 10.1093/ofid/ofaf475

**Published:** 2025-08-08

**Authors:** Samora Té, Edvaldo Das Neves, Ane Pellicer, Ioanna Broumou, Julia Zerebinski, Ana Santos-Reis, Taís Nóbrega de Sousa, Anna Färnert, Adalberto Santos, Maria de Jesus Santos, Jose Pedro Gil, Dinora Lopes

**Affiliations:** Science and Community Support Service (SACC), Institute of Hygiene and Tropical Medicine, Nova University of Lisbon, Lisbon, Portugal; Global Health and Tropical Medicine, GHTM, Associate Laboratory in Translation and Innovation Towards Global Health, LA-REAL, Instituto de Higiene e Medicina Tropical, IHMT, Universidade NOVA de Lisboa, UNL, Rua da Junqueira 100, 1349-008 Lisboa, Portugal; Science and Community Support Service (SACC), Institute of Hygiene and Tropical Medicine, Nova University of Lisbon, Lisbon, Portugal; Division of Infectious Diseases, Department of Medicine, Solna, Karolinska Institutet, Stockholm, Sweden; Department of Infectious Diseases, Karolinska University Hospital, Stockholm, Sweden; Division of Infectious Diseases, Department of Medicine, Solna, Karolinska Institutet, Stockholm, Sweden; Department of Infectious Diseases, Karolinska University Hospital, Stockholm, Sweden; Science and Community Support Service (SACC), Institute of Hygiene and Tropical Medicine, Nova University of Lisbon, Lisbon, Portugal; Global Health and Tropical Medicine, GHTM, Associate Laboratory in Translation and Innovation Towards Global Health, LA-REAL, Instituto de Higiene e Medicina Tropical, IHMT, Universidade NOVA de Lisboa, UNL, Rua da Junqueira 100, 1349-008 Lisboa, Portugal; Host Microbe Division, Department of Microbiology, Tumor and Cell Biology (MTC), Karolinska Institutet, Stockholm, Sweden; Molecular Biology and Malaria Immunology Research Group, Instituto René Rachou, Fundação Oswaldo Cruz (FIOCRUZ), Belo Horizonte, Brazil; Division of Infectious Diseases, Department of Medicine, Solna, Karolinska Institutet, Stockholm, Sweden; Department of Infectious Diseases, Karolinska University Hospital, Stockholm, Sweden; Instituto Nacional de Saúde Pública, Ministério da Saúde, São Tomé, São Tomé and Príncipe; Instituto Nacional de Saúde Pública, Ministério da Saúde, São Tomé, São Tomé and Príncipe; Global Health and Tropical Medicine, GHTM, Associate Laboratory in Translation and Innovation Towards Global Health, LA-REAL, Instituto de Higiene e Medicina Tropical, IHMT, Universidade NOVA de Lisboa, UNL, Rua da Junqueira 100, 1349-008 Lisboa, Portugal; Host Microbe Division, Department of Microbiology, Tumor and Cell Biology (MTC), Karolinska Institutet, Stockholm, Sweden; Science and Community Support Service (SACC), Institute of Hygiene and Tropical Medicine, Nova University of Lisbon, Lisbon, Portugal; Global Health and Tropical Medicine, GHTM, Associate Laboratory in Translation and Innovation Towards Global Health, LA-REAL, Instituto de Higiene e Medicina Tropical, IHMT, Universidade NOVA de Lisboa, UNL, Rua da Junqueira 100, 1349-008 Lisboa, Portugal; Tropical Medicine Unit, Institute of Hygiene and Tropical Medicine, Nova University of Lisbon, Lisbon, Portugal

**Keywords:** ACT, amodiaquine, drug resistance, *pfmdr1*, SNP

## Abstract

Artesunate–amodiaquine has been the first-line treatment for uncomplicated malaria in São Tomé and Príncipe since 2005, following many decades of chloroquine usage. We evaluated the recent dynamics in prevalence of the *Plasmodium falciparum* amodiaquine-resistance-associated *pfmdr1* 86Y/Y184/1246Y haplotype, by analyzing infections from the 2020–2022 period and comparing them with historical data. The results suggest that the introduction of artesunate–amodiaquine led to a trajectory of single nucleotide polymorphism (SNP) successive accretion in a pre-existing environment of *pfcrt* 76T near fixation, leading to the emergence of a dominant 86Y/Y184/1246Y haplotype. Our data support increased vigilance of artesunate–amodiaquine performance in São Tomé and Príncipe.

The island nation of São Tomé and Príncipe (STP) is one of the most geographically isolated malaria settings in Africa, a status offering a near-unique opportunity for elimination success in Equatorial Africa. Accordingly, STP has a well-established malaria control program both in terms of vector control and case management. The latter is based on the use of artesunate–amodiaquine (ASAQ) for case management as a directly observed treatment and a 28-day mandatory treatment surveillance follow-up. These efforts, associated with a solid vector control plan, resulted in a significant decrease in malaria incidence, allowing the country to transit from a holoendemic setting status by the start of the century toward a case incidence of <1% during the last decade [1]. Unfortunately, this progression has stagnated since ∼2015, following the pattern of many other African regions [2].

The efficacy of ASAQ, the first-line artemisinin-based combination therapy (ACT) for the management of uncomplicated malaria in STP since 2007, has not been formally tested for >10 years, with only a limited appraisal performed providing pilot data [3]. This scarcity of data leaves room for hypothesizing if the slower progression towards pre-elimination witnessed in the last years is related to decreased ASAQ performance. Worryingly, long-known field ex vivo data prior to ASAQ implementation in the country already identified *Plasmodium falciparum* parasites showing significantly elevated AQ IC_50_ [4].

Mutations in the *P. falciparum* multidrug resistance 1 gene (*pfmdr1*), namely the 86Y/Y184/1246Y haplotype—particularly in the context of *pfcrt* 76T—is the most established set of molecular markers of AQ efficacy [5]. The isolated characteristics of STP offer an opportunity to study the prevalence evolution of these alleles, with a minimum of external interference due to the relatively rare importation of malaria cases. Accordingly, we herein investigated the trajectory of this set of 4 critical single nucleotide polymorphisms (SNPs) during the 2020–2022 period while comparing it with historical data, including the transit from chloroquine monotherapy to ASAQ. We confirm the near fixation of *pfcrt* 76T, as well as a recent selection of the *pfmdr1* Y184 and 1246Y markers, toward the establishment of the AQ-resistance-associated 86Y/Y184/1246Y as the dominant haplotype in STP.

## METHODS

Filter paper samples with preserved blood spots were obtained from 283 microscopically confirmed uncomplicated malaria cases included in the surveillance activities of the São Tomé and Príncipe National Malaria Elimination Program, Centro Nacional de Endemias.

All samples were irreversibly anonymized, and the study was approved by the IHMT-ITQB Ethics Committee (ref. 16.22) and the Swedish Ethical Review Authority (ref. 2017/499-32). These samples refer to the 2020–2022 period (2020, n = 95; 2021, n = 102; 2022, n = 86) with the majority of them (73.7%) resourced from the Àgua Grande District, which includes the metropolitan area of São Tomé, which contributes with over 50% of the nation's annual malaria incidence.

DNA was extracted using QIAamp DNA Mini kit (Qiagen) according to the manufacturer's instructions. *Plasmodia* species determination was performed by PCR targeting the 18 ssrRNA gene [6]. Genotyping of *pfmdr1* N86Y, Y184F, and D1246Y was done using Polymerase Chain Reaction-Restriction Fragment length polymorphism (PCR-RFLP) methods as previously published [5 ]; *pfcrt* K76T was analyzed by direct PCR amplicon Sanger sequencing. All oligonucleotide primers and amplification conditions are described in Supplementary Table 1.

Previous reports point to the STP parasite populations as characterized by low genetic diversity [1], consistent with the isolation of the island, as well as the multi-decade 4-aminoquinoline antimalarial pressure. In order to confirm this scenario in our samples, we evaluated the *pfmsp2* gene diversity, choosing samples from the year 2021 for analysis. Genotyping was performed by nested PCR with fluorescently labeled primers targeting the 2 FC27 and IC allelic types of *msp2*, respectively (Supplementary Table 1), followed by fragment sizing by capillary electrophoresis, as described previously [7], with slight modifications. Fragment sizes were binned within ±1 bp to account for measurement error. Genomic DNAs from the 3D7, Dd2, and HB3 *P. falciparum* strains were used as positive controls.

Data analysis and visualization for *msp2* genotyping was performed in R Studio version 4.3.2.

Historical SNP frequency data were resourced from references [1, 4, 8]. For the latter 2, original data were available and used, as this represented works originally performed at the Institute of Hygiene and Tropical Medicine.

Fisher exact test was used for analyzing differences between proportions. Continuous variable distribution normality was checked with the Kolmogorov–Smirnov test. Median and averaged differences were evaluated with the Kruskal–Wallis test and the *t*-test, respectively.

## RESULTS

All samples were found to represent *P. falciparum* mono-infections. Analysis of *pfcrt* was successful in 82% of the samples (Supplementary Figure 1), confirming the historical dominance of the 76T allele (main 72–76 haplotype: CVIET) with prevalence levels above 95%, a likely legacy from chloroquine resistance that was carried over toward the ASAQ period. Also, the K76 allele was unambiguously found, albeit in low frequencies (<5%), in contrast to previous studies during the time when CQ was the first-line antimalarial in STP [4, 8].

**Figure 1.**
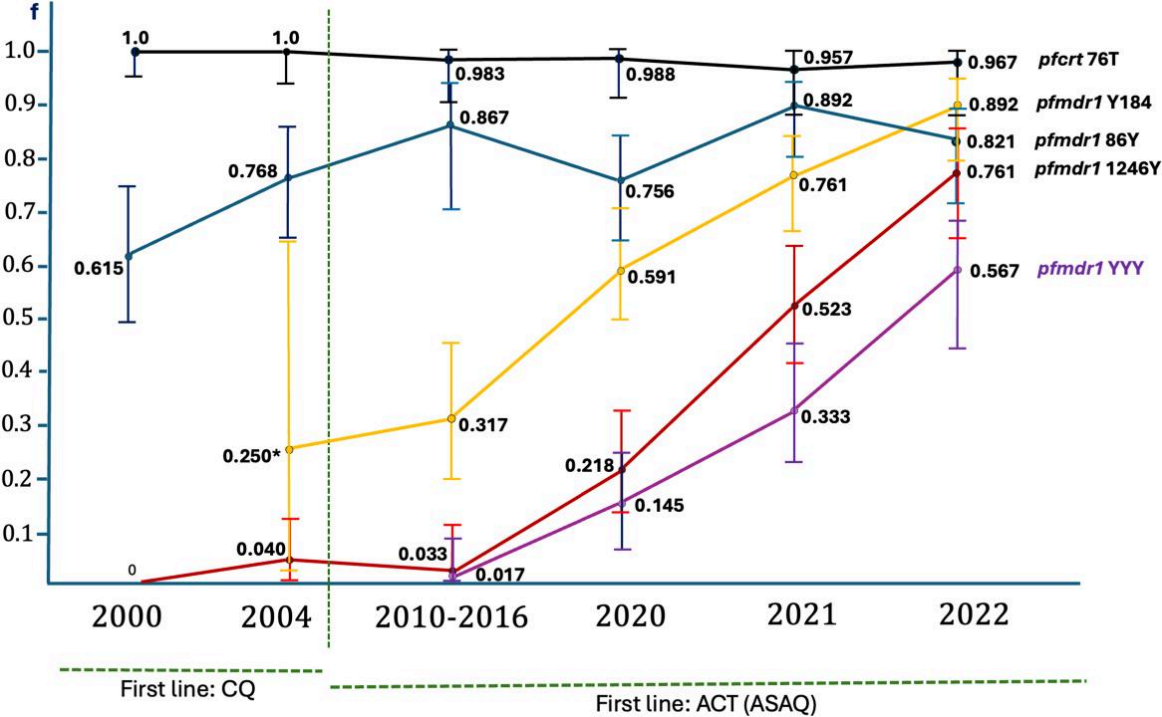
Drug-resistance-associated gene polymorphisms timeline in São Tomé and Príncipe. Only genotypes classified as “pure” were herein included. Similar RFLP and amplicon Sanger sequencing methods were used for all the reports. With the exception of the 2010–2016 period [1], the remaining works (2000 [n = 60], 2004 [n = 69], and present data) were previously performed in the same research unit [4, 8]. Vertical lines represent confidence interval 95%. *2004 Y184F data are only based on 8 samples, albeit 6 of them being pure 184F infections, suggesting a high frequency of this allele during the chloroquine usage period.

The *pfmdr1 N*86Y analysis was successful in 94.7% of the samples (Supplementary Figure 1). Consistent with a 4-aminoquinoline-saturated background, the 86Y allele was present in a stable high prevalence, consistently above 70%, with no significant variation during the studied 2020–2022 period, as well as when comparing the period before and after the transition from CQ to ASAQ (Figure 1). The analysis of the Y184F and D1246Y SNP, both documented as specifically linked to AQ resistance, had an overall success rate of 89.8% and 86.2%, respectively (Supplementary Figure 1). The resistance-associated alleles Y184 and 1246Y showed an intriguing pattern for increased prevalence along time, with a significant acceleration during the 2020–2022 window. By the end of this period, Y184 had reached similar frequencies to the 86Y allele, starting from 30% levels according to pre-2020 historical data (*P* < .001). The D1246Y SNP also showed a notable increase from below 5% prevalence before our 2020 data, toward >70% by 2022 (*P* < .001; Figure 1). Importantly, the rise of these alleles is reflected in parallel by the emergence of the AQ resistance *pfmdr1* 86Y/Y184/1246Y haplotype [5, 9], which kept increasing its frequency along the analyzed timeline among our STP sample set.

**Figure 2.**
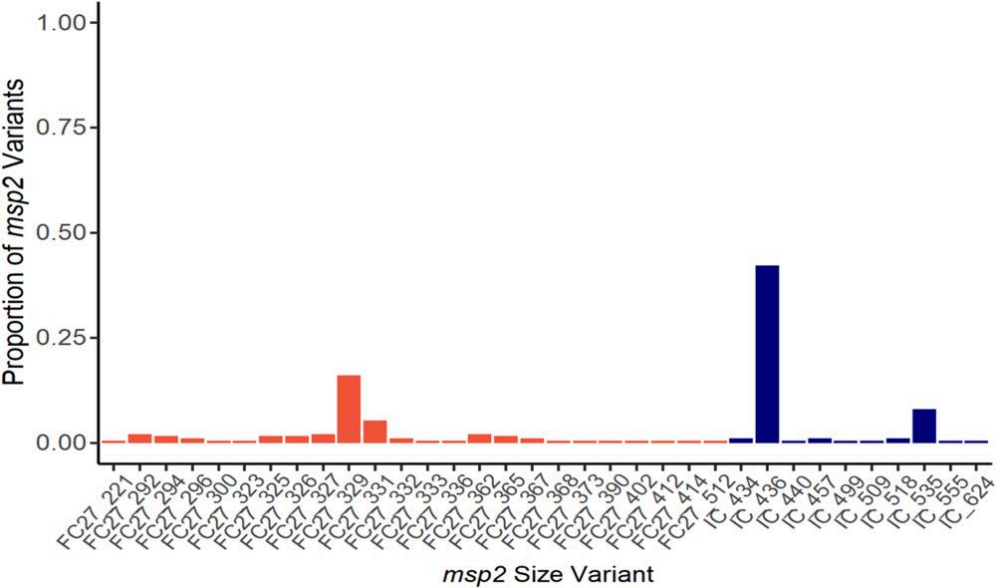
Proportion of *pfmsp2* allele size variants genotyped (FC27 and IC families) in *P. falciparum* samples from 2021, highlighting the dominance of the FC27_329 bp, IC_436 bp, and IC 535 bp alleles.

The clear dominance of 4-aminoquinoline-resistance-associated alleles in a potential AQ-driven process led us to confirm previous data of a relatively low diversity of São Tomé parasite populations, indicative of selection bottleneck events [1]. Genotyping of the highly polymorphic genetic marker *pfmsp2* confirmed the low diversity of the parasite population. In 102 samples from 2021, the observed multiplicity of infection was 2 (95% confidence interval 1.96–2.16). IC-type *msp2* was detected in 98 (96.1%) of the samples, while 73 samples (72%) harbored an FC27-type *msp2*. Three *msp2* variants were highly prevalent in the samples from 2021: IC 436 (435–437 bp) detected in 79 (77.5%), FC27 329 (328–330 bp) in 30 (29%), and IC 535 (534–536 bp) in 15 of 102 (15%) genotyped samples; covering 42%, 16%, and 8% of all 187 detected *pfmsp2* size variants, respectively (Figure 2). The high prevalence of 2 *pfmsp2* allelic variants corroborates the low diversity of local *P. falciparum* populations.

## DISCUSSION

STP switched almost directly from chloroquine toward ASAQ, keeping the local parasite populations under an over 70 years unbroken period of 4-aminoquinoline pressure. This is primarily reflected in the very high (≥95%) prevalence of the chloroquine-resistance *pfcrt* 76T mutation, that kept unchanged upon the transition to ACT. This intense drug pressure is likely to have long-term shaped the structure of these parasite populations, leading to very high frequencies of *pfcrt* 76T, an allele known to be critical for both chloroquine and AQ resistance. This bottleneck is supported by our *pfmsp2* genotyping, which reinforces data from previous works pointing to a low level of diversity among STP *P. falciparum* parasites [1].

In this background of *pfcrt* 76T fixation, the time domain patterns of critical *pfmdr1* N86Y, Y184F, and D1246Y SNP prevalence are more informative, as we can see a successive increase in the frequency of these alleles, in a suggested sequential way. The *pfmdr1* 86Y, a well-established secondary factor in chloroquine resistance, seems to have firstly stabilized at high frequencies (>75%) during the 2010–2016 period [1], apparently followed by the parallel increase in 2020–2022 for both Y184 and 1246Y. The latter allele was originally detected in São Tomé during the times of chloroquine use [10], being later confirmed until 2016 to be present in frequencies below 10% in the Água Grande region [1, 4], before experiencing a fast rise in the herein analyzed years.

The overall selection dynamics suggest a long-term trajectory toward the establishment of the 86Y/Y184/1246Y haplotype, known to be associated with decreased sensitivity to AQ and ASAQ clinical failure [5, 11]. This pattern of haplotype refinement is in a manner reminiscent of the previously observed evolution of *pfdhfr* SNPs linked to pyrimethamine–sulfadoxine (SP) resistance [12]. There, the pathway requires the obligatory pre-establishment of the S108N allele, which allows then the addition of position N51I and C59R, as well as mutations in *pfdhps*, leading to a significant decrease in SP efficacy. We suggest that *pfcrt* 76T has an equivalent foundational role of 108N with SP, now for ASAQ. A robust prevalence of *pfmdr1* 86Y is another legacy of chloroquine monotherapy times, which is also important for AQ, and its active metabolite desethylamodiaquine (DEAQ). The observed patterns of in vivo long-term selection of the 86Y/Y184/1246Y haplotype are consistent with highly controlled gene manipulation data, as N86 to 86Y allele exchange leads to a 2-fold IC_50_ increase for both drugs [13]. As for D1246Y, exchanging the alleles from D to Y did not give a significant difference in CQ IC_50_ [14], but rather an ∼2-fold increase for both AQ and DEAQ [11]. This is compatible with our timeline observations, where this allele stayed at very low levels during the time of extensive CQ use [3, 10], being subsequently selected upon the introduction of ASAQ. Finally, Y184F has been determined as having more of a supportive function [14]. All these changes decrease the efficiency of the coded P-glycoprotein (Pgp), either by blocking the efflux of the substrate (86Y) or by increasing the energy needed for the transport-linked conformation transition of the Pgp [15]. In both cases, it is believed that this lowers the capacity of the transporter to accumulate AQ and DEAQ in the food vacuole, the target compartment for these antimalarials, enhancing the parasite survival odds.

The key remaining question concerns the present efficacy of ASAQ in STP. No properly sized efficacy trial has been conducted in the country since the introduction of the ASAQ combination. Considering data from AQ-based therapies performed in East Africa at the time of transition to ACT, it is to note that the cure rates in the subset of recruited patients with 86Y/Y184/1246Y infections were frequently below the WHO 90% threshold [5, 9]. To a certain extent, this group of patients carrying AQ refractory infections will be treated with a partial AS monotherapy, further endangering the future of the latter antimalarial, and the overall future of ACT in the country. Under this perspective, our present findings reinforce the urgent need for the performance of an extensive ASAQ direct observed treatment efficacy trial in STP.

In conclusion, our data points for a parasite population significantly selected by multi-year exposure to ASAQ are likely in the path toward AQ resistance. Under a more positive perspective, and acknowledging the collateral sensitivity relation of amino alcohol quinolines with 4-aminoquinolines [16, 17] (Supplementary Table 2), we expect for the present malaria parasites in STP to be highly susceptible to lumefantrine, and hence to AL therapy. A switch to this ACT will likely constitute a feasible near-future strategy if ASAQ is confirmed to be losing efficacy in the country.

## Supplementary Material

ofaf475_Supplementary_Data

## Supplementary Data

Supplementary materials are available at *Open Forum Infectious Diseases* online. Consisting of data provided by the authors to benefit the reader, the posted materials are not copyedited and are the sole responsibility of the authors, so questions or comments should be addressed to the corresponding author.
