# Supplementary material for: Trajectory of Plasmodium falciparum Molecular Markers of Amodiaquine Resistance in São Tomé and Príncipe
Source: Open Forum Infect Dis. 2025 Aug 8;12(8):ofaf475. doi: 10.1093/ofid/ofaf475 (PMC12378731; doi:10.1093/ofid/ofaf475)
Supplement: ofaf475_Supplementary_Data [file ofaf475_supplementary_data.docx]

**Supplementary material**

| **Target** | **Oligonucleotide primers** | **Amplification** |
| --- | --- | --- |
| *pfmdr1* N86Y - first | fw:5’AAGAGGTTGAAAAAGAGTTGAAC 3’  rev: 5’ATTTCGTACCAATTCCTGAACT 3’ | [94˚C, 30s + 56˚C, 30s + 60˚C, 30s] x45 cycles |
| *pfmdr1* N86Y - nest | fw: 5’AGAGTACCGCTGAATTATTTAG 3’  rev: 5’CCTGAACTCACTTGTTCTAAAT 3’ | [94˚C, 30s + 47˚C, 30s + 68˚C, 1min] x40 cycles |
| *pfmdr1* Y184F first | fw: 5’ AAGAGGTTGAAAAAGAGTTGAAC 3’  rev: 5’ CTTATTACATATGACACCACAAAC 3’ | [92˚C, 20s + 53˚C, 30s + 68˚C, 1.15min] x40 cycles |
| *pfmdr1* Y184F nest | fw: 5’ACATATGCCAGTTCCTTTTTAGGTTAAT 3’  rev: 5’ AACATAAAGTAACGGAAAAACGCAA 3’ | [92˚C, 20s + 63˚C, 30s + 68˚C, 30s] x10 cycles + [92˚C, 20s + 60˚C, 30s + 68˚C, 30s] x 10 cycles + [92˚C, 20s + 58˚C, 30s + 68˚C, 30s] x 20 cycles |
| *pfmdr1* D1246Y first | fw: 5’CTACAGCAATCGTTGGAGAAA 3’  rev: 5’GCTCTAGCTATAGCTATTCTC 3’ | [92˚C, 30s + 55˚C, 30s + 68˚C, 30s] x 45 cycles |
| *pfmdr1* D1246Y nest | fw: 5’AACCAATCTGGATCTGCAGAAGA 3’  rev: 5’ACATCTTCCAATGTTGCATCTTCT 3’ | [92˚C, 30s + 58˚C, 30s + 68˚C, 45s] x 20 cycles + [92˚C, 30s + 57˚C, 30s + 68˚C, 1min] x 20 cycles |
| *pfcrt* K76T -first | fw: 5’CAAGAAGGAAGTAAGTATCCAAAATGG 3’  rev: 5’GTAGTTCTTGTAAGACCTATGAAGGC 3’ | [94˚C, 30s + 56˚C, 30s + 60˚C, 1min] x 45 cycles |
| *pfcrt* K76T -nest | fw: 5’GCAAAAATGACGAGCGTTATAGAG 3’  rev: 5’ CTGAACAGGCACTAACATGGATATAGC 3’ | [94˚C, 30s + 47˚C, 30s + 68˚C, 1min] x 45 cycles |
| *pfmsp2 first (*full ORF) | fw:5'ATGAAGGTAATTAAAACATTGTCTATTATA 3'  rev: 5'CTTTGTTACCATCGGTACATTCTT3' | [95˚C, 5 min] + [58˚C, 2 min + 72˚C, 2 min + 94˚C, 1 min] x 25 cycles + [58˚C, 2 min] + [72˚C, 5 min] |
| *pfmsp2* nest  *(targeting the two pfmsp2 families)* | fw (FC27): 5' AATACTAAGAGTGTAGGTGCARAT GCTCCA 3'  rev 6-FAM™ (FC27): 5' TTTTATTTGGTGCATTGCCAGAACTTGAAC 3' ;  fw (IC): 5' AGAAGTATGGCAGAAAGTAAKCCTY CTACT 3',  rev VIC^®^(IC) 5' GATTGTAATTCGGGGGATTCAGT TTGTTCG 3' | [95˚C, 5 min] + [61˚C, 2 min + 72˚C, 2 min + 94˚C, 1 min] x 23 cycles + [61˚C, 2 min] + [72˚C, 5 min] |

**Table S1** – PCR amplification oligonucleotide primers and amplification conditions.

| **Gene/allele** | **AQ/DEAQ** | **LUM** | **AS** |
| --- | --- | --- | --- |
| *pfmdr1* N86 | **-** | **+** | **+** |
| *pfmdr1* 86Y | **+** | **-** | **-** |
| *pfmdr1* Y184 | **+** | **-** | **-** |
| *pfmdr1* 184F | **-** | **+** | **+** |
| *pfmdr1* D1246 | **-** | **+** | **+** |
| *pfmdr1* 1246Y | **+** | **-** | **-** |
| *pfcrt* K76 | **-** | **+** | **+** |
| *pfcrt* 76T | **+** | **-** | **-** |

**Table S2** – *pfmdr1* and *pfcrt* SNPs under focus and their association with *P*. *falciparum* response to major ACT drugs. (+): increased resistance, (-): sensitive. AQ: amodiaquine, LUM: lumefantrine, ATS: artesunate.


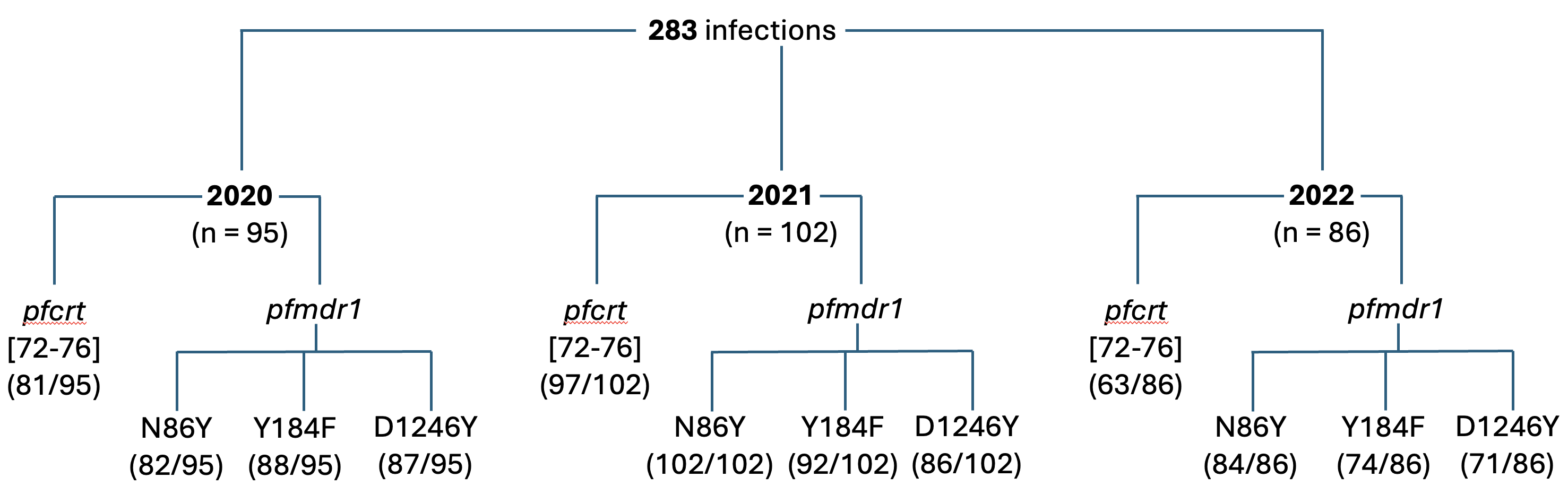


**Figure S1** – Genotype success rates per target sequence analysed in 283 *P. falciparum* infections.
